# The impact of the COVID-19 pandemic on the continuity of transfusion care for thallasemic patients: a case report

**DOI:** 10.11604/pamj.2021.38.33.26591

**Published:** 2021-01-13

**Authors:** Sabah Bouhou, Mohammed Benajiba, Azlarab Masrar

**Affiliations:** 1 National Centre for Blood Transfusion and Hematology, Rabat, Morocco,; 2 Hematology research team, Hematology laboratory, Faculty of Medicine and Pharmacy, Mohammed V University, Rabat, Morocco,; 3 Central Hematology laboratory, Ibn Sina University Hospital Center, Rabat, Morocco

**Keywords:** Thalassemia, COVID-19, pandemic, transfusion, care-continuity, case report

## Abstract

The COVID-19 pandemic could have a major impact on the capacity of health systems to continue the delivery of essential health service. While health systems around the world are being challenged by increasing demand for care of COVID-19 patients, it is critical to maintain preventive and curative services, especially for the most vulnerable populations such people living with chronic conditions like thallasemics. In this context and since the start of the SARS-CoV-2 health crisis, the National Blood Transfusion Center of Morocco has ranked among its priorities the need to maintain transfusion management for chronic polytransfused patients, particularly those with thalassemia. We report in this paper, the case of a thallasemic patient whose transfusion management was disrupted by the restrictive measures introduced by the Moroccan authorities and for which the National Blood Center of Morocco provided effective support.

## Introduction

The COVID-19 pandemic could have a major impact on the capacity of health systems to continue the delivery of essential health services [1]. While health systems around the world are being challenged by increasing demand for care of COVID-19 patients, it is critical to maintain preventive and curative services, especially for the most vulnerable populations such people living with chronic conditions like thallasemics [1]. In the same way, this pandemic was considered by international scientific societies to have an impact on blood donation and transfusion activities at the level of transfusion establishments on an international scale. Efforts must, therefore, be made at the level of the transfusion centers to ensure proper management of this health crisis at SARS-CoV-2 [2-4]. The National Blood Center of Morocco (MNBC) as a responsible for the implementation of the Moroccan Ministry of Health's policy on blood transfusion [5], has ensured since the outbreak of the SARS-CoV-2 pandemic a health, epidemiological and scientific watch in order to ensure good management of this health crisis at the level of the Moroccan Blood Transfusion Centers. Several measures have been adopted by the Moroccan national blood centre to ensure effective support for donors, clinicians, patients and for Regional Blood Transfusion Centers. The main objective is to ensure the availability and safety of blood products for patients in need including polytransfused patients like patients with thalassemia.

## Patient and observation

The Moroccan National Blood Centre (MNBC) was contacted on 22^th^ March 2020 by the president of the association of patients with thalassemia and hemoglobinopathy in the Al Hoceima region to request the Moroccan National Blood Centre intervention about a thalassemia patient. This is a 15-year-old Beta-thalassemia youngster patient living in the Al Hoceima region but who is monitored since the discovery of her illness in the Paediatric Hemato Oncology Department (PHOD) of the Ibn Sina University Hospital in Rabat where she receives blood transfusions according to a well-codified transfusion protocol pre-established by her attending physician. This is a patient who is already immunologically known to have two antibodies: anti-Jka and anti-S. The last transfusion received at the paediatric hemato oncology department dates back to 03^rd^ March 2020 where the patient had received three iso phenotypically iso group red blood cells compatible with her current immunological status. The test for irregular agglutinins was negative.

The patient had an appointment on 23^th^ March 2020 in Rabat to receive her usual transfusion but to which she could not go given the measures taken on 21^th^ March 2020 by the Moroccan authorities in connection with the situation health related to COVID-19 and concerning the ban on travel between cities in Morocco. But her mother reports that she is very tired and its biological assessment shows a severe normochromic normocytic anaemia with a haemoglobin level at: 5.8g/dl. After receiving all the necessary information, the Moroccan National Blood Centre began the process of organizing the transfusion management of the patient on her city of residence at the Mohammed V Hospital of Al Hoceima city. After an effective intervention of several actors the patient was hospitalized and was transfused on March 25 2020 with two red blood cells products, compatible with its current immunological status, prepared by the Regional blood centre of Oujda who is qualified for the preparation of qualified blood products for thalasemic patients in the region. The red blood cells products were transported to Al Hoceima city with the assistance of the local health authorities and delivered by the Regional Blood Transfusion Centre of Al Hoceima after having ensured leucocyte free products. Note that the Al Hoceima city is at 457 kilometres far from Rabat city and at 260 kilometers far from Oujda city.

On April 06, following a state of tiredness, a biological assessment was done who reported severe normochromic normocytic anemia with a hemoglobin level at: 6.2g/dl. The National Blood Centre has started the procedure of regulation and coordination between the Regional Blood Centre of Oujda, the Regional Blood Centre of Al Hoceima and the health authorities of the region and the patient was hospitalized and transfused on April 10 with 03 red blood cell pellets, compatible with its current immunological status, prepared by Regional Blood Centre of Oujda and delivered by the Regional Blood centre of Al Hoceima. On April 28 the Moroccan national blood center was contacted again regarding the patient who was clinically impaired and with a blood count showing a hemoglobin level at: 5.9g/dl. Faced with the suspicion of transfusion inefficiency, the National Blood Centre contacted the responsible of the pediatric department of the Oujda University hospital centre in order to hospitalize the patient for a complete biological and immunohematological assessment.

On May 01, the patient was hospitalized, taken care of and put on corticosteroid treatment. The erythrocyte genotyping carried out at the Regional Blood Centre of Rabat had found the patient to be Fya-. Therefore, the patient may have developed anti-Fya antibody during subsequent transfusions and this could explain her transfusion inefficiency. This information was helpful for the Blood Centre of Oujda to prepare appropriate blood units for this patient. So, after clinical improvement and stabilization, the patient was transfused with 4 red blood cell pellets compatible with her new immuno hematological status: Jka-, S-and Fya-. These blood products were prepared and delivered by the Immunohematology laboratory of Oujda Regional Blood Centre. The patient left the hospital after 15 days of hospitalization and presented a good clinical and biological evolution with a hemoglobin level at 11g/dl.

On June 03, the patient had a follow-up appointment at the pediatric department of the University Hospital of Oujda. A biological assessment shows a hemoglobin level at 9.5g/dl. She has been transfused with three red blood cells products with good biological recovery. From July 01, the patient's medical file was transferred from the Rabat University Hospital to the Oujda University Hospital where she is regularly monitored and taken care of according to a transfusion protocol well established by her new attending physician. To date, no complaint has been received by the National Blood Centre about the management of this thallasemic patient. We present in Table 1 a chronological summary of the care provided to the patient with the effective intervention of the Moroccan National Blood Centre.

**Table 1:** chronological summary of the care provided to the patient with the effective intervention of the MNBC

Date	Insured support
03/25/2020	Two pellets of red blood cells Jka- and S-, compatible with her immunological state, prepared by Regional Blood Centre of Oujda and delivered by Regional Blood Centre of Al Hoceima
04/10/2020	Three red blood cell pellets, compatible with her current immunological state, prepared by the Regional Blood Centre Oujda and delivered by the Regional Blood Centre of Al Hoceima
05/01/2020	Hospitalization at the Oujda University Hospital for transfusion inefficiency
	Put on corticosteroid treatment with complete assessment
	Detection of irregular agglutinins positive
	Discovery that the patient was Fya- following genotyping carried out at Regional Blood Centre of Rabat
	Transfused after stabilization with 4 red blood cell pellets, compatible with her current immunological state (Jka-, S- and Fya-) prepared by the Regional Blood Centre of Oujda
	Patient discharged after stabilization with a good clinical and biological evolution
06/03/2020	The patient had an appointment at the University Hospital of Oujda and was transfused with three compatible red blood cells prepared and delivered by the Regional Blood Centre of Oujda
From July 1	The patient's medical file transferred to Oujda University Hospital with regular monitoring and support provided by the Oujda Regional Blood Centre and the Oujda University Hospital according to a well-identified protocol
	No complaint about this patient has been received by the Moroccan National Blood Centre from this date

## Discussion

Thalassemia is the consequence of a synthesis imbalance between the α and β chains of haemoglobin. It´s a hereditary haemolytic anaemia, which presents a problem of public health in Morocco because of their frequency and the difficulties of their care [6]. Patients with thalassemia are regularly transfused in order to maintain a pre-transfusion haemoglobin level of at least 9-10 gr/dl. This high level of transfusion is necessary to correct the anaemia and allow a level of correct activity but also to suppress residual thalassemia erythropoiesis responsible for some complications: extra medullary haematopoiesis, increased risk of thrombosis and pulmonary arterial hypertension [7]. Treatment is instituted for life and relies on a monthly transfusion and iron chelation, but the distance to care and the high cost of treatment are the main challenges faced by patients and their families. It is a very serious condition both in terms of health, economically and socially [7].

In Morocco, since 1990, the paediatric haematology and oncology service at the children's hospital at Ibn Sina University Hospital in Rabat city was the only structure that has provided regular and scheduled follow-up for thalassemia patients [8]. But in 2011 the Moroccan health ministry launched a program to fight hereditary diseases of hemoglobin, particularly thalassemia [8]. This program aims for: (i) the generalization by 2012 of care to all patients. (ii) The establishment of a budget for the purchase of blood products and all drugs for poor patients. (iii) The creation of medical care structures in different regions of the country to avoid long trips to Rabat. Following that, since 2012, the Moroccan National Blood Center has ensured the qualification of 08 blood transfusion centers for the management of illnesses with hemoglobinopathies in 08 Moroccan Cities: Rabat, Casablanca, Fez, Oujda, Marrakech, Tangier, Agadir and El Jadida. In addition, the Moroccan National Blood Center has established the preparation of phenotyped and leukocyte-free blood products for all thallasemics through the purchase each year from 2012 of leukoreduction filters and phenotyping reagents. Over the years, the National Blood Center has ranked among its priorities ensuring the availability and proximity of quality blood products to these patients and continued provided adequate management of thallasemics patients.

In the current COVID-19 pandemic context, most scientifics societies stipulate that in thalassemia there is no need to change the care and the transfusion rules except in the event of a major shortage of red blood cell concentrates, which is not currently the case, or well in the situation where the coming of the patient to his transfusion site is considered to be too high risk transmission of COVID-19 or impossible due to restrictive displacement measures implemented by local authorities [7]. In this context, Intezar Mehdi *et al*. stipulate that COVID-19 pandemic has posed significant challenges for children with cancers and blood disorders such hemoglobinopathies. They report that interruption or postponement of treatment would be an additional challenge either due to COVID-19 infection or inability to reach the centres for treatment due to lockdowns [9].

Dimitrios Farmakis *et al*. reported that the COVID-19 pandemic represents a significant challenge for haemoglobinopathy patients, their families and their attending physicians. They present a statement who summarizes the key challenges concerning the management of haemoglobinopathies, with particular focus on patients with either transfusion-dependent or non-transfusion-dependent thalassaemia [10]. They illustrate that adaptation of thalassaemia care during the present and potential future similar pandemics requires on one hand the strengthening of existing and creation of new communication channels between healthcare professionals and patients and on the other the promotion of a modified patient pathway for access to care including visits to medical facilities [10].

Jamie Hartmann-Boyce *et al*. report that disruption of care, diversion of healthcare resources, and interruptions to medical supplies can all impact patients with long term conditions (LTCs) during national emergencies [11]. They report that though much of the literature comes from natural disasters, the sparse literature on pandemics as they relate to LTC management has identified how indirect health impacts can further increase morbidity and mortality [11]. Causes of this phenomenon include diversion or depletion of resources, and decreased access to routine care resulting from inability to travel, fear, or other factors. For example, during the 2014 West Africa Ebola epidemic, lack of routine care for other conditions is estimated to have contributed to 10,600 additional deaths in Guinea, Liberia, and Sierra Leone [11].

**Figure 1:**
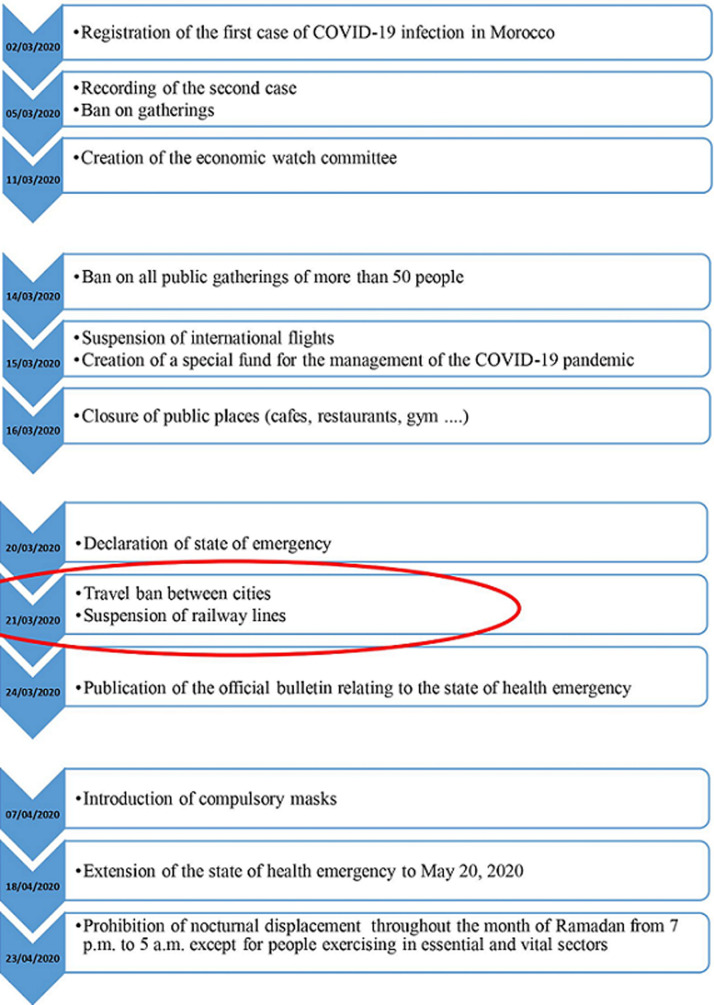
chronogram of the major measures adopted by the kingdom of Morocco according to the evolution of the epidemiological situation with COVID-19

Bruno Fattizo *et al*. reported the strategies of progressive systematic reorganisation of hematologic care in a large public university hospital in Milan (Lombardy) during the first 6 weeks of the COVID-19 pandemic [12]. They observed that urgent transfusions were guaranteed, and transfusion-dependent patients adapted their schedules to fit with donor availability (1 unit per week instead of 2 units per 14 days), because the transfusion centre experienced a marked reduction in donors. With regard to hospitalization, admissions to the haematology and transplant wards showed a decrease at week 1; thereafter, the limited number of admissions did not allow for definitive conclusions to be made [12]. Like other countries, Morocco has implemented several measures early on to deal with the COVID-19 pandemic and to allow good management of this health crisis [13]. The main measures are shown in Figure 1. In this context, following the measure taken at March 21 2020 as the ban on travel between cities, our patient had a problem of moving from her place of residence at the level of the city of AL Hoceima to the city of Rabat where she is followed and benefits from a transfusion treatment according to a codified protocol established by her treating specialist.

This situation could have impacted the continuity of her care and could have prevented the patient from continuing to receive the transfusion treatment from which she was benefiting. The rapid reactivity of the Moroccan National Blood Centre allowed early and adapted management of this patient's case. In addition, this effective and efficient intervention of the National Blood Centre and the regulatory and coordination mission that it ensured between the various actors involved in this medical file such as staff of the regional blood centres, the regional and local health authorities and the thalassemia association allowed the patient to continue to receive her therapeutic protocol in the best conditions and with the appropriate blood products. This support took into consideration the evolution and the worsened of her immunological status with the discovery of a new information relating to the erythrocytic genotyping which showed that the patient is Fya- besides that she is already known to have two antibodies: anti-JKa and anti-S.

## Conclusion

During national emergencies and pandemics like COVID-19 pandemic, health care services and supply chains may be disrupted. Health care resources may be limited and a focus on controlling a pandemic will shift focus from other areas [11]. Health systems must put in place appropriate recommendations and programs to ensure continuity of care for patients with long-term conditions such as hemoglobinopathies. The National Blood Center of Morocco has demonstrated a great reactivity and a great adaptability since the beginning of the epidemic according to the evolution of scientific data to ensure the availability and safety of blood products to patients. Through a strategy of listening, sharing and active communication, the staff of the various transfusion centers demonstrated exceptional dedication, an incomparable commitment during this difficult period and have shown effective support for patients in need. This case report is a concrete example of the dedication, the involvement and the commitment demonstrated by the transfusion staff towards patients in need of appropriate and continuous transfusion care. This support is provided through the provision of quality blood products adapted to the evolution of their clinical situations.
